# Supplementary material for: Maternal dietary fat during lactation shapes single nucleus transcriptomic profile of postnatal offspring hypothalamus in a sexually dimorphic manner in mice
Source: Nat Commun. 2024 Mar 16;15:2382. doi: 10.1038/s41467-024-46589-x (PMC10944494; doi:10.1038/s41467-024-46589-x)
Supplement: Supplementary file 1 — Supplementary Information [file 41467_2024_46589_MOESM1_ESM.pdf]

Supplementary Information for

Maternal dietary fat during lactation shapes single nucleus transcriptomic profile of postnatal offspring hypothalamus in a sexually dimorphic manner in mice

Yi Huang[1,$,†], Anyongqi Wang[1,2†], Wenjiang Zhou[3], Baoguo Li[1,4], Linshan Zhang[3], Agata M. Rudolf[1], Zengguang Jin[5], Catherine Hambly[6], Guanlin Wang[3, †,*], and John R. Speakman[1,5,6,7,*]

[1]State Key Laboratory of Molecular Developmental Biology, Institute of Genetics and Developmental Biology, Chinese Academy of Sciences, Beijing,100101, China.

[2] University of Chinese Academy of Sciences, Beijing,101408, China.

[3] Shanghai Key Laboratory of Metabolic Remodeling and Health, Institute of Metabolism and Integrative Biology, Centre for Evolutionary Biology, Fudan University, Shanghai, 200438, China.

[4] Tianjian Laboratory of Advanced Biomedical Sciences, Zhengzhou University, Zhengzhou, China, 450001, China.

[5] Shenzhen Key Laboratory of Metabolic Health, Center for Energy Metabolism and Reproduction, Shenzhen Institutes of Advanced Technology, Chinese Academy of Sciences, Shenzhen, 518055, China.

[6] School of Biological Sciences, University of Aberdeen, Aberdeen, AB24 3FX, UK .

[7] China Medical University, Shenyang, Liaoning,110122, China

[†] These authors contribute equally

[$] Current address: Broad Institute of MIT and Harvard, Metabolism Program, Cambridge, MA, 02142, USA.

*Corresponding authors: GW: guanlin_wang@fudan.edu.cn; JRS: j.speakman@abdn.ac.uk

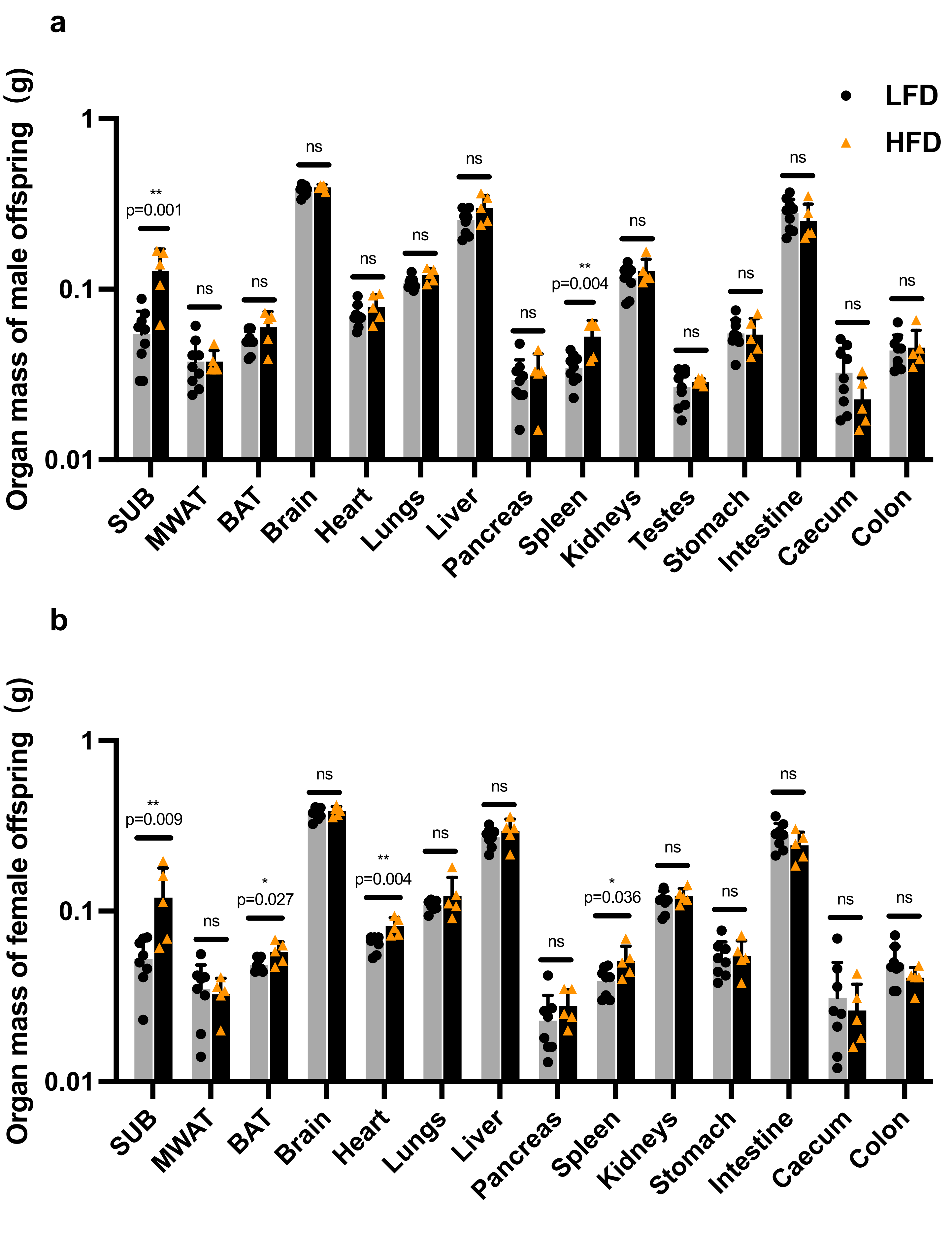
a
Organ mass of male offspring (g)
1
0.1
0.01
LFD
HFD
**
p=0.001
**
p=0.004
ns
SUB
MWAT
BAT
Brain
Heart
Lungs
Liver
Pancreas
Spleen
Kidneys
Testes
Stomach
Intestine
Caecum
Colon
b
Organ mass of female offspring (g)
**
p=0.009
*
p=0.027
**
p=0.004
*
p=0.036
ns

Supplementary Fig. 1. Organ masses in postnatal day 15 (P15) male and female offspring raised by mothers fed on diets with low fat and high fat during lactation, related to Figure 1.

(a) Organ masses in subcutaneous fat (SUB), mesenteric fat (MWAT), brown adipose tissue (BAT), brain, heart, lungs, liver, pancreas, spleen, kidneys, testes, stomach, intestine, caecum and colon in male P15 offspring.

(b) Organ masses in subcutaneous fat (SUB), mesenteric fat (MWAT), brown adipose tissue (BAT), brain, heart, lungs, liver, pancreas, spleen, kidneys, stomach, intestine, caecum and colon in female P15 offspring. Values are means± s.d. *p value* by one-way ANOVA. * represents $p < 0.05$, ** represents $p < 0.01$. ns represents no significance between comparisons. Sample sizes for male and female offspring raised by mother fed LFD were 9, those fed LFD were 5. Source data are provided as a Source Data file.

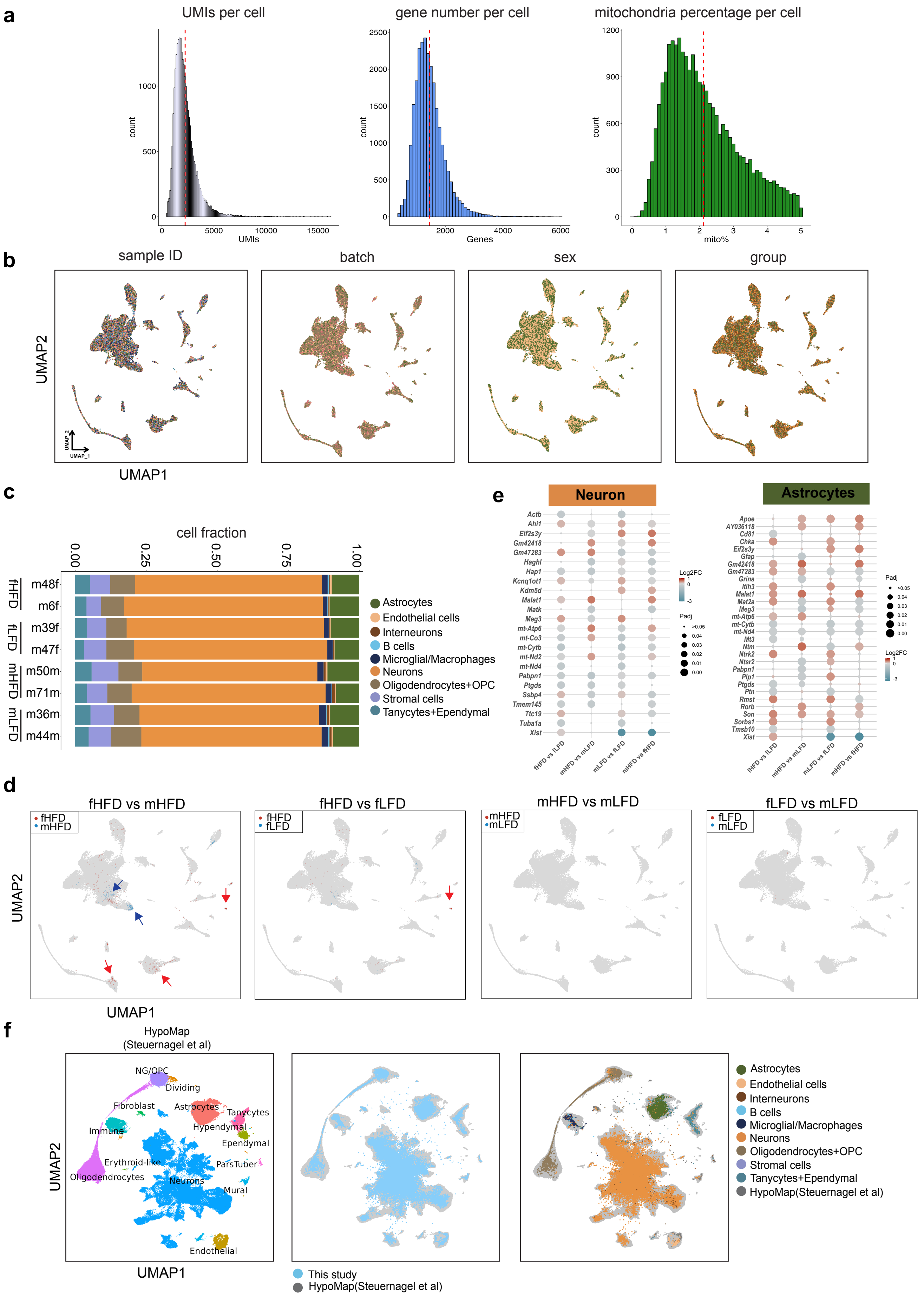
a
UMIs per cell
count
UMIs
gene number per cell
Genes
mitochondria percentage per cell
mito%
b
sample ID
batch
sex
group
UMAP2
UMAP1
c
cell fraction
0.00
0.25
0.50
0.75
1.00
fHFD
m48f
m6f
fLFD
m39f
m47f
mHFD
m50m
m71m
mLFD
m36m
m44m
Astrocytes
Endothelial cells
Interneurons
B cells
Microglial/Macrophages
Neurons
Oligodendrocytes+OPC
Stromal cells
Tanycytes+Ependymal
e
Neuron
Astrocytes
Actb
Ahi1
Eif2s3y
Gm42418
Gm47283
Haghl
Hap1
Kcnq1ot1
Kdm5d
Malat1
Matk
Meg3
mt-Atp6
mt-Co3
mt-Cytb
mt-Nd2
mt-Nd4
Pabpn1
Ptgds
Ssbp4
Tmem145
Ttc19
Tuba1a
Xist
Apoe
AY036118
Cd81
Chka
Gfap
Grina
Itih3
Mat2a
Mt3
Ntm
Ntrk2
Ntsr2
Plp1
Ptn
Rmst
Rorb
Son
Sorbs1
Tmsb10
Log2FC
Padj
fHFD vs fLFD
mHFD vs mLFD
mLFD vs fLFD
mHFD vs fHFD
d
fHFD vs mHFD
fHFD vs fLFD
mHFD vs mLFD
fLFD vs mLFD
fHFD
mHFD
fLFD
mLFD
UMAP2
UMAP1
f
HypoMap
(Steuernagel et al)
NG/OPC
Dividing
Fibroblast
Astrocytes
Tanycytes
Hypendymal
Immune
Ependymal
Erythroid-like
ParsTuber
Oligodendrocytes
Neurons
Mural
Endothelial
UMAP2
UMAP1
This study
HypoMap(Steuernagel et al)
Astrocytes
Endothelial cells
Interneurons
B cells
Microglial/Macrophages
Neurons
Oligodendrocytes+OPC
Stromal cells
Tanycytes+Ependymal
HypoMap(Steuernagel et al)

Supplementary Fig. 2. A comprehensive map of mouse hypothalamus at P15, related to Figure 2.

(**a**) QC matrices of the single nucleus transcriptomics analysis. Histograms of the distribution of the UMIs per cell, gene number per cell and mitochondrial percentage per cell.

(**b**) Uniform Manifold Approximation and Projections (UMAPs) showing the results after batch correction, colored by sample ID, batch, sex and experimental groups.

(**c**) Barplot of the cell proportions of key lineages from each individual (indicated as mouse sampleID).

(**d**) Differential abundance analysis (DAseq) shows the enrichment of abundance of cells from the full dataset when compares fHFD vs mHFD, fLFD vs mLFD, mHFD vs mLFD and fHFD vs fLFD. Colored dots represent the enrichment of abundance in different groups.

(**e**) Dotplot of the top/bottom 5 DEGs ordered by Log2 fold change (Log2FC) between the comparisons of fHFD vs fLFD, mHFD vs mLFD, mLFD vs fLFD and mHFD vs fHFD in neuron and astrocytes. *p value* was adjusted by Bonferroni correction.

(**f**) Comparison of the single nuclei captured in our study to previously published study by projecting our dataset (this study, light blue) onto the HypoMap dataset (Steuernagel *et al*, light grey).

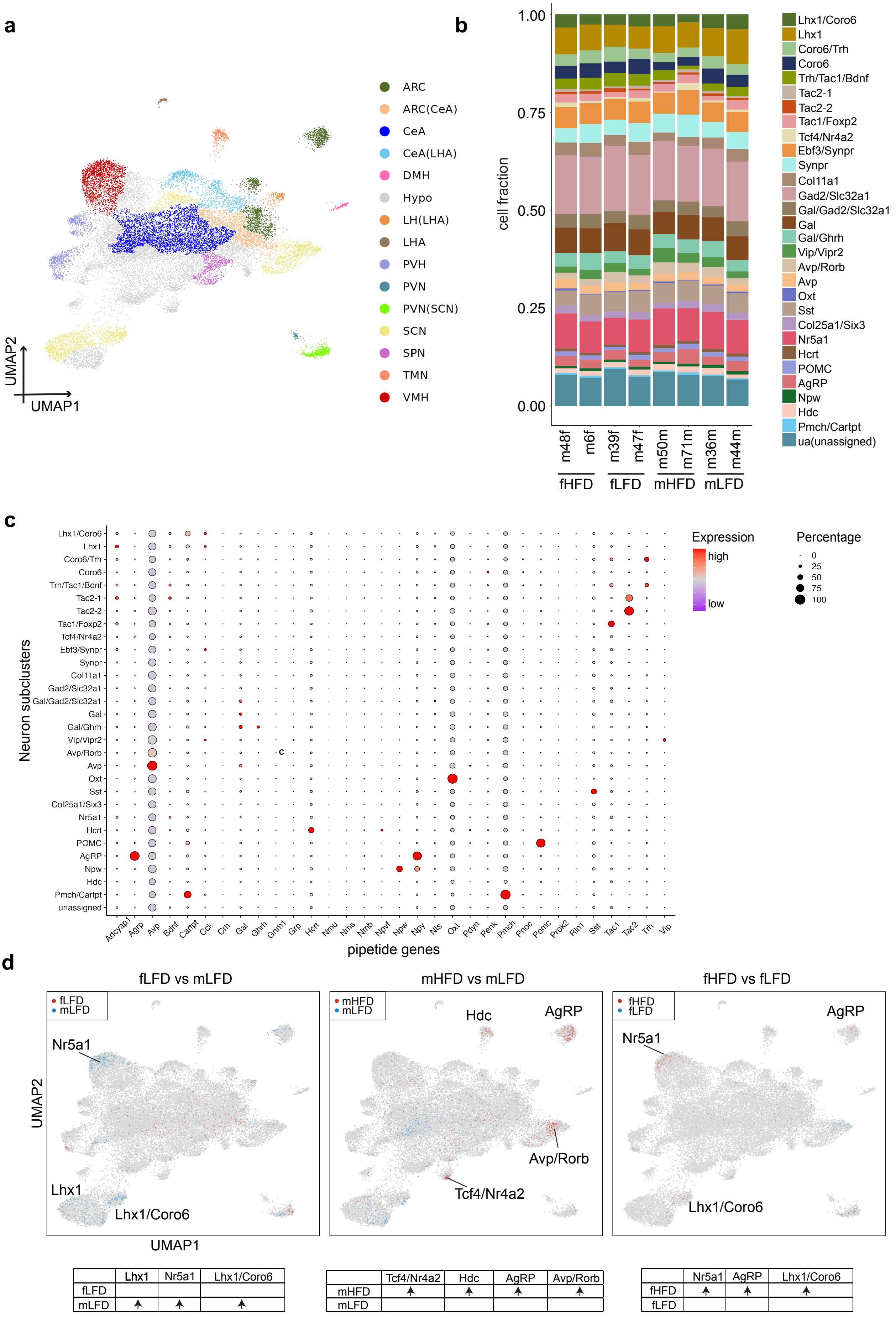
a
ARC
ARC(CeA)
CeA
CeA(LHA)
DMH
Hypo
LH(LHA)
LHA
PVH
PVN
PVN(SCN)
SCN
SPN
TMN
VMH
UMAP2
UMAP1
b
cell fraction
1.00
0.75
0.50
0.25
0.00
m48f
m6f
m39f
m47f
m50m
m71m
m36m
m44m
fHFD
fLFD
mHFD
mLFD
Lhx1/Coro6
Lhx1
Coro6/Trh
Coro6
Trh/Tac1/Bdnf
Tac2-1
Tac2-2
Tac1/Foxp2
Tcf4/Nr4a2
Ebf3/Synpr
Synpr
Col11a1
Gad2/Slc32a1
Gal/Gad2/Slc32a1
Gal
Gal/Ghrh
Vip/Vipr2
Avp/Rorb
Avp
Oxt
Sst
Col25a1/Six3
Nr5a1
Hcrt
POMC
AgRP
Npw
Hdc
Pmch/Cartpt
ua(unassigned)
c
Neuron subclusters
Lhx1/Coro6
Lhx1
Coro6/Trh
Coro6
Trh/Tac1/Bdnf
Tac2-1
Tac2-2
Tac1/Foxp2
Tcf4/Nr4a2
Ebf3/Synpr
Synpr
Col11a1
Gad2/Slc32a1
Gal/Gad2/Slc32a1
Gal
Gal/Ghrh
Vip/Vipr2
Avp/Rorb
Avp
Oxt
Sst
Col25a1/Six3
Nr5a1
Hcrt
POMC
AgRP
Npw
Hdc
Pmch/Cartpt
unassigned
Adcyap1 Agrp Avp Bdnf Cartpt Cck Crh Gal Ghrh Gnrh1 Grp Hcrt Nmu Nms Nmb Npvf Npw Npy Nts Oxt Pdyn Penk Pmch Pnoc Pomc Prok2 Rln1 Sst Tac1 Tac2 Trh Vip
pipetide genes
Expression
high
low
Percentage
0
25
50
75
100
d
fLFD vs mLFD
fLFD
mLFD
Nr5a1
Lhx1
Lhx1/Coro6
mHFD vs mLFD
mHFD
mLFD
Hdc
AgRP
Avp/Rorb
Tcf4/Nr4a2
fHFD vs fLFD
fHFD
fLFD
Nr5a1
AgRP
Lhx1/Coro6
UMAP2
UMAP1
| Lhx1 | Nr5a1 | Lhx1/Coro6
fLFD | | |
mLFD | ↑ | ↑ | ↑
| Tcf4/Nr4a2 | Hdc | AgRP | Avp/Rorb
mHFD | ↑ | ↑ | ↑ | ↑
mLFD | | | |
| Nr5a1 | AgRP | Lhx1/Coro6
fHFD | ↑ | ↑ | ↑
fLFD | | |

Supplementary Fig. 3. Heterogeneity of the neuronal subpopulations, related to Figure3.

(**a**) UMAPs of the hypothalamic regions by different colors. Cells that not assigned to any specific region were coloured in gray.

(**b**) Barplot of the cell proportions of neuronal subpopulations from fHFD, fLFD, mHFD and mLFD groups.

(**c**) Dot plots of the expression of canonical neuropeptide and neurotransmitter genes in neuronal subpopulations.

(**d**) Differential abundance analysis (DAseq) shows the enrichment of abundance of cells from different neuronal subpopulations when compares fLFD vs mLFD, mHFD vs mLFD and fHFD vs fLFD. Colored dots represent the enrichment of abundance in different groups. (Red – mHFD; Blue – mLFD)

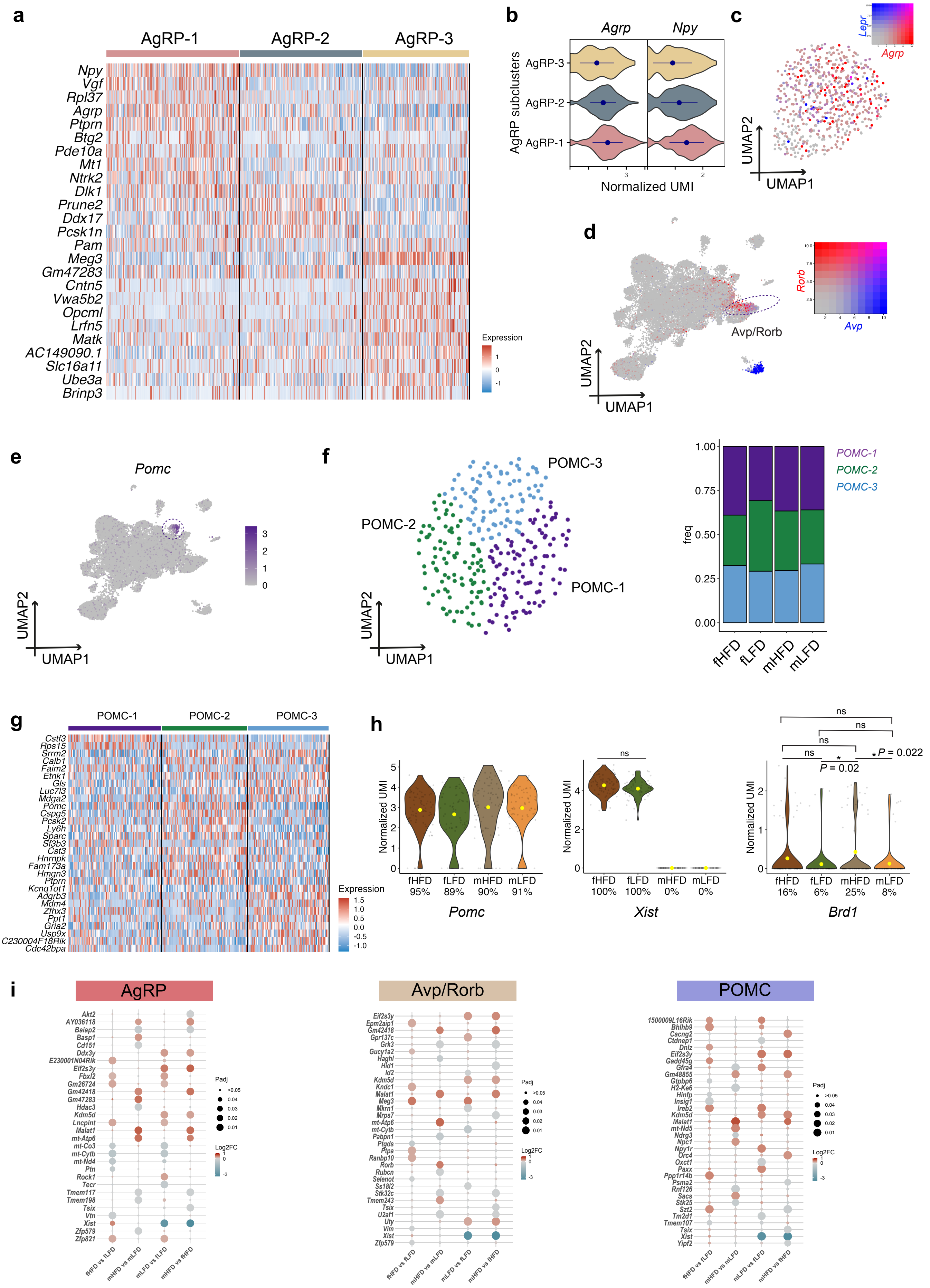
a
AgRP-1
AgRP-2
AgRP-3
Npy
Vgf
Rpl37
Agrp
Ptprn
Btg2
Pde10a
Mt1
Ntrk2
Dlk1
Prune2
Ddx17
Pcsk1n
Pam
Meg3
Gm47283
Cntn5
Vwa5b2
Opcml
Lrfn5
Matk
AC149090.1
Slc16a11
Ube3a
Brinp3
Expression
b
Agrp
Npy
AgRP subclusters
AgRP-3
AgRP-2
AgRP-1
Normalized UMI
c
Lepr
Agrp
UMAP2
UMAP1
d
Rorb
Avp
Avp/Rorb
UMAP2
UMAP1
e
Pomc
UMAP2
UMAP1
f
POMC-3
POMC-2
POMC-1
UMAP2
UMAP1
freq
POMC-1
POMC-2
POMC-3
fHFD
fLFD
mHFD
mLFD
g
POMC-1
POMC-2
POMC-3
Cstf3
Rps15
Srrm2
Calb1
Faim2
Etnk1
Gls
Luc7l3
Mdga2
Pomc
Cspg5
Pcsk2
Ly6h
Sparc
Sf3b3
Cst3
Hnrnpk
Fam173a
Hmgn3
Ptprn
Kcnq1ot1
Adgrb3
Mdm4
Zfhx3
Ppt1
Gria2
Usp9x
C230004F18Rik
Cdc42bpa
Expression
h
Normalized UMI
fHFD 95%
fLFD 89%
mHFD 90%
mLFD 91%
Pomc
ns
fHFD 100%
fLFD 100%
mHFD 0%
mLFD 0%
Xist
ns
ns
ns
ns
*
P = 0.02
* P = 0.022
fHFD 16%
fLFD 6%
mHFD 25%
mLFD 8%
Brd1
i
AgRP
Avp/Rorb
POMC
Padj
Log2FC
fHFD vs fLFD
mHFD vs mLFD
mLFD vs fLFD
mHFD vs fHFD

Supplementary Fig. 4. Heterogeneity of AgRP/Npy and POMC neurons, related to Figure 4.

(**a**) Heatmap of top 10 differentially expressed genes in each AgRP subcluster.

(**b**) Violin plots of the expression of *Agrp* and *Npy* in each AgRP subcluster.

(**c**) *Lepr* and *Agrp* co-expression is shown on UMAP of AgRP subclusters. Red colour represents the expression of *Agrp* and *Lepr* for blue, blended colour in pink to purple as well as pink represents the co-expression of two genes. Grey represents for no expression.

(**d**) *Avp* and *Rorb* co-expression is shown on UMAP of neuronal subclusters. Red colour represents the expression of *Rorb* and *Avp* for blue, blended colour in pink to purple as well as pink represents the co-expression of two genes. Grey represents for no expression.

(**e**) UMAP of canonical marker genes for POMC neurons (*Pomc*) on the of neuronal subclusters.

(**f**) Unsupervised clustering of extracted POMC neurons identified 3 distinct subtypes and barplot shows the composition of POMC subclusters from fHFD, fLFD, mHFD and mLFD groups.

(**g**) Heatmap of the top 10 differentially expressed genes in each POMC subcluster.

(**h**) Violin plots of the expression of *Pomc*, *Xist* and *Brd1* genes in four groups in POMC neurons. * represents $P < 0.05$ for Wilcoxon test.

(**i**) Dotplot of the top/bottom 5 DEGs ordered by Log2FC between the comparisons of fHFD vs fLFD, mHFD vs mLFD, mLFD vs fLFD and mHFD vs fHFD in key neuronal subpopulations. *p value* was adjusted by Bonferroni correction.

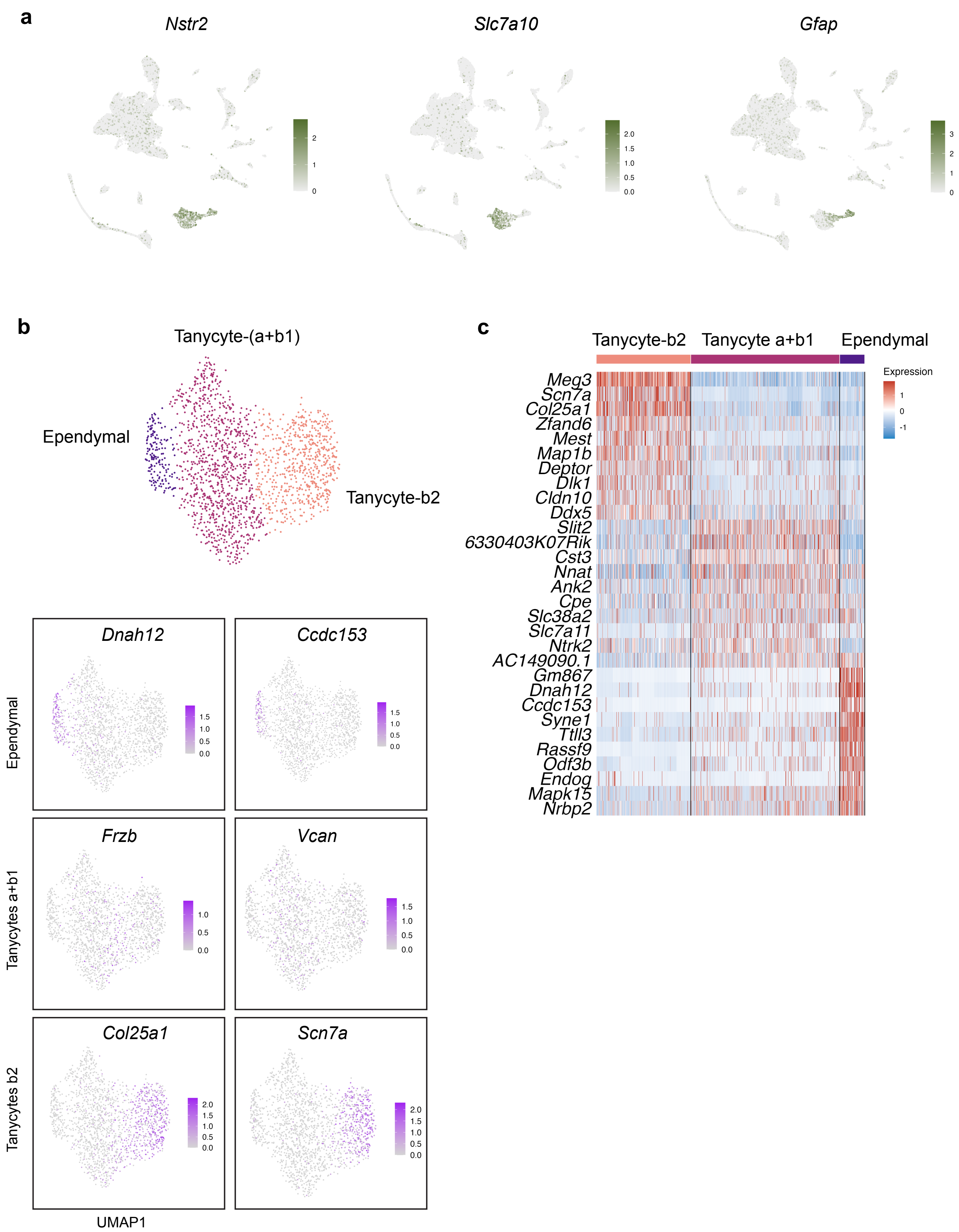
a
Nstr2
Slc7a10
Gfap
b
Tanycyte-(a+b1)
Ependymal
Tanycyte-b2
Dnah12
Ccdc153
Frzb
Vcan
Col25a1
Scn7a
Ependymal
Tanycytes a+b1
Tanycytes b2
UMAP1
c
Tanycyte-b2
Tanycyte a+b1
Ependymal
Expression
Meg3
Scn7a
Col25a1
Zfand6
Mest
Map1b
Deptor
Dlk1
Cldn10
Ddx5
Slit2
6330403K07Rik
Cst3
Nnat
Ank2
Cpe
Slc38a2
Slc7a11
Ntrk2
AC149090.1
Gm867
Dnah12
Ccdc153
Syne1
Ttll3
Rassf9
Odf3b
Endog
Mapk15
Nrbp2

Supplementary Fig. 5. Heterogeneity of non-neuronal subclusters, related to Figure 5.

(**a**) Expressions of canonical astrocyte marker genes *Nstr*, *Slc7a10* and *Gfap* on UMAP.

(**b**) Unsupervised clustering of extracted tanycytic and ependymal cells identified 3 distinct subtypes and the expression of canonical marker genes *Dnah12*, *Ccdc153* (Ependymal), *Fzrb, Vcan* (tanycytes a+b1) and *Col25a1, Scn7a* (tanycytes b2) on UMAP.

(**c**) Heatmap of the top 10 differentially expressed genes in each tanycytic and ependymal subcluster.

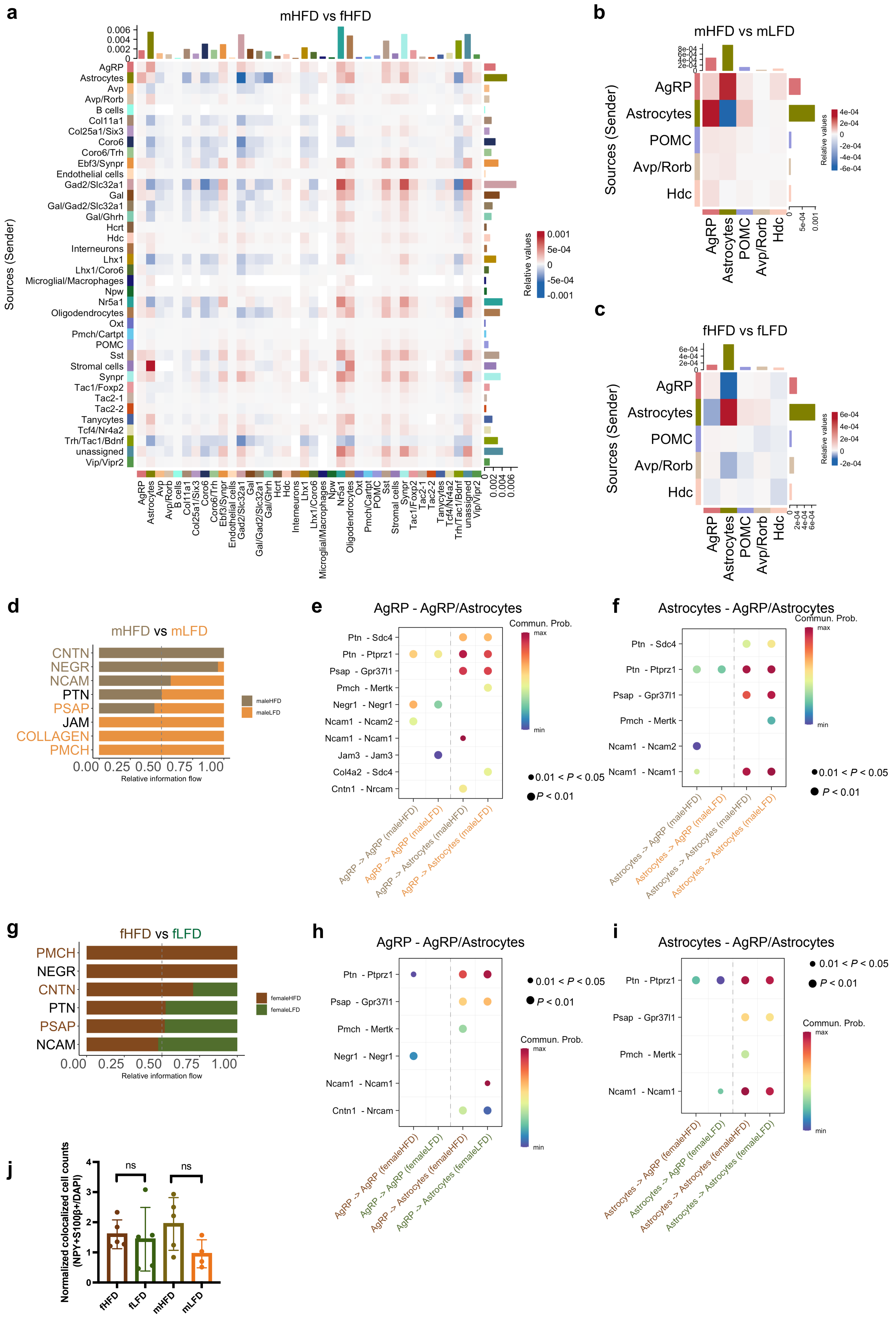
a
mHFD vs fHFD
Sources (Sender)
Relative values
0.001
5e-04
0
-5e-04
-0.001
0.006
0.004
0.002
0
AgRP
Astrocytes
Avp
Avp/Rorb
B cells
Col11a1
Col25a1/Six3
Coro6
Coro6/Trh
Ebf3/Synpr
Endothelial cells
Gad2/Slc32a1
Gal
Gal/Gad2/Slc32a1
Gal/Ghrh
Hcrt
Hdc
Interneurons
Lhx1
Lhx1/Coro6
Microglial/Macrophages
Npw
Nr5a1
Oligodendrocytes
Oxt
Pmch/Cartpt
POMC
Sst
Stromal cells
Synpr
Tac1/Foxp2
Tac2-1
Tac2-2
Tanycytes
Tcf4/Nr4a2
Trh/Tac1/Bdnf
unassigned
Vip/Vipr2
b
mHFD vs mLFD
Sources (Sender)
AgRP
Astrocytes
POMC
Avp/Rorb
Hdc
Relative values
4e-04
2e-04
0
-2e-04
-4e-04
-6e-04
8e-04
6e-04
4e-04
2e-04
0
5e-04
0.001
c
fHFD vs fLFD
Sources (Sender)
AgRP
Astrocytes
POMC
Avp/Rorb
Hdc
Relative values
6e-04
4e-04
2e-04
0
-2e-04
d
mHFD vs mLFD
CNTN
NEGR
NCAM
PTN
PSAP
JAM
COLLAGEN
PMCH
maleHFD
maleLFD
0.00 0.25 0.50 0.75 1.00
Relative information flow
e
AgRP - AgRP/Astrocytes
Commun. Prob.
max
min
Ptn - Sdc4
Ptn - Ptprz1
Psap - Gpr37l1
Pmch - Mertk
Negr1 - Negr1
Ncam1 - Ncam2
Ncam1 - Ncam1
Jam3 - Jam3
Col4a2 - Sdc4
Cntn1 - Nrcam
0.01 < P < 0.05
P < 0.01
AgRP -> AgRP (maleHFD)
AgRP -> AgRP (maleLFD)
AgRP -> Astrocytes (maleHFD)
AgRP -> Astrocytes (maleLFD)
f
Astrocytes - AgRP/Astrocytes
Commun. Prob.
max
min
Ptn - Sdc4
Ptn - Ptprz1
Psap - Gpr37l1
Pmch - Mertk
Ncam1 - Ncam2
Ncam1 - Ncam1
0.01 < P < 0.05
P < 0.01
Astrocytes -> AgRP (maleHFD)
Astrocytes -> AgRP (maleLFD)
Astrocytes -> Astrocytes (maleHFD)
Astrocytes -> Astrocytes (maleLFD)
g
fHFD vs fLFD
PMCH
NEGR
CNTN
PTN
PSAP
NCAM
femaleHFD
femaleLFD
0.00 0.25 0.50 0.75 1.00
Relative information flow
h
AgRP - AgRP/Astrocytes
Ptn - Ptprz1
Psap - Gpr37l1
Pmch - Mertk
Negr1 - Negr1
Ncam1 - Ncam1
Cntn1 - Nrcam
0.01 < P < 0.05
P < 0.01
Commun. Prob.
max
min
AgRP -> AgRP (femaleHFD)
AgRP -> AgRP (femaleLFD)
AgRP -> Astrocytes (femaleHFD)
AgRP -> Astrocytes (femaleLFD)
i
Astrocytes - AgRP/Astrocytes
0.01 < P < 0.05
P < 0.01
Ptn - Ptprz1
Psap - Gpr37l1
Pmch - Mertk
Ncam1 - Ncam1
Commun. Prob.
max
min
Astrocytes -> AgRP (femaleHFD)
Astrocytes -> AgRP (femaleLFD)
Astrocytes -> Astrocytes (femaleHFD)
Astrocytes -> Astrocytes (femaleLFD)
j
Normalized colocalized cell counts (NPY+S100β+/DAPI)
ns
ns
0
1
2
3
4
fHFD
fLFD
mHFD
mLFD

Supplementary Fig. 6. Cell-cell interaction on the mouse P15 hypothalamus atlas, related to Figure 6.

(**a**) Overall interactive signaling changes on all the key lineages and neuron subpopulations indicating that astrocytes are the key hotspot of the interactions with AgRP neurons between mHFD and fHFD groups.

(**b**) & (**c**) Overall interactive signaling changes on key neuronal subpopulations and top scoring non-neuronal populations between (**b**) mHFD and mLFD groups as well as between (**c**) fHFD and fLFD groups.

(**d**) The identified key pathways of the interactive signaling between mHFD and mLFD groups.

(e) & **(f)** The identified key up-regulated and down-regulated signaling ligand-receptor pairs between the interactions of AgRP neurons and astrocytes in mHFD and mLFD groups.

(**g**) The identified key pathways of the interactive signaling between fHFD and fLFD groups.

(**h**) & (**i**) The identified key up-regulated and down-regulated signaling ligand-receptor pairs between the interactions of AgRP neurons and astrocytes in fHFD and fLFD groups.

(**j**) Normalized cell counts of colocalization of Npy+ (represents AgRP/Npy neurons) and S100β+ (to stain astrocytes), normalization calculated by Npy+S100β+/DAPI+ cells. Biologically independent animals for fHFD group were 5, for fLFD group were 5, for mHFD group were 5, for mLFD group were 4. *p value* by two-sided Student's *t*-test. Values are means ±s.d. ns represents no significance between comparisons. Source data are provided as a Source Data file.
