# Supplementary material for: Maternal dietary fat during lactation shapes single nucleus transcriptomic profile of postnatal offspring hypothalamus in a sexually dimorphic manner in mice
Source: Nat Commun. 2024 Mar 16;15:2382. doi: 10.1038/s41467-024-46589-x (PMC10944494; doi:10.1038/s41467-024-46589-x)
Supplement: Supplementary file 3 — Description of Additional Supplementary Information [file 41467_2024_46589_MOESM3_ESM.pdf]

Description of Additional Supplementary Files

Supplementary Data 1. Physiological measurements of mouse. *p value* by one-way ANOVA or RM-GLM in BW, FI and EI analysis. *p value* by GLM adjusted for BW in MEI, DEE, MEO and dissection analysis.

Supplementary Data 2. QC matrics, of full hypothalamus object and neuron subpopulations.

Supplementary Data 3. Cluster size of full hypothalamus object and neuron subclusters.

Supplementary Data 4. Marker genes of major clusters and neuronal subpopulations as well as DEGs/GO between different maternal dietary groups. *p value* was adjusted by Bonferroni correction.

Supplementary Data 5. Genes that related to pseudotime trajectory in astrocytes.

Supplementary Data 6. Significant ligand-receptor pairs in four maternal dietary groups
