# Supplementary material for: Maternal dietary fat during lactation shapes single nucleus transcriptomic profile of postnatal offspring hypothalamus in a sexually dimorphic manner in mice
Source: Nat Commun. 2024 Mar 16;15:2382. doi: 10.1038/s41467-024-46589-x (PMC10944494; doi:10.1038/s41467-024-46589-x)
Supplement: Supplementary file 10 — Reporting Summary [file 41467_2024_46589_MOESM10_ESM.pdf]

nature portfolio

Corresponding author(s): John R. Speakman; Guanlin Wang

Last updated by author(s): Feb 18, 2024

Reporting Summary

Nature Portfolio wishes to improve the reproducibility of the work that we publish. This form provides structure for consistency and transparency in reporting. For further information on Nature Portfolio policies, see our Editorial Policies and the Editorial Policy Checklist.

Statistics

For all statistical analyses, confirm that the following items are present in the figure legend, table legend, main text, or Methods section.

n/a	Confirmed	
☐	☒	The exact sample size (*n*) for each experimental group/condition, given as a discrete number and unit of measurement
☐	☒	A statement on whether measurements were taken from distinct samples or whether the same sample was measured repeatedly
☐	☒	The statistical test(s) used AND whether they are one- or two-sided<br>*Only common tests should be described solely by name; describe more complex techniques in the Methods section.*
☐	☒	A description of all covariates tested
☐	☒	A description of any assumptions or corrections, such as tests of normality and adjustment for multiple comparisons
☐	☒	A full description of the statistical parameters including central tendency (e.g. means) or other basic estimates (e.g. regression coefficient) AND variation (e.g. standard deviation) or associated estimates of uncertainty (e.g. confidence intervals)
☐	☒	For null hypothesis testing, the test statistic (e.g. *F*, *t*, *r*) with confidence intervals, effect sizes, degrees of freedom and *P* value noted<br>*Give P values as exact values whenever suitable.*
☒	☐	For Bayesian analysis, information on the choice of priors and Markov chain Monte Carlo settings
☒	☐	For hierarchical and complex designs, identification of the appropriate level for tests and full reporting of outcomes
☒	☐	Estimates of effect sizes (e.g. Cohen's *d*, Pearson's *r*), indicating how they were calculated

Our web collection on statistics for biologists contains articles on many of the points above.

Software and code

Policy information about availability of computer code

Data collection	No software was used for data collection in this study.
Data analysis	Single nucleus RNA sequencing raw data were processed with Cell Ranger (version 4.0.0) with default parameters. for each sample and mapped to the mm10-3.0.0 genome to generate unique molecular identifiers(UMI) expression matrices. Seurat (4.1.0) was performed for normalization (SCTransform), dimensionality reduction, clustering, integration and differentially expressed gene analysis. Symphony (0.1.1) was performed for reference mapping. DAseq (1.0.0) was performed for differential abundance analysis. Monocle3 (V3 0.2.3) were performed for trajectory analysis. ClusterProlifier (v4.9.1) were performed for pathway enrichment analysis. Cellchat was performed for cell cell interaction prediction. Imaris (version 9.8.0) was applied for the cell counting and co-localization analysis. Statistical analysis were performed using SPSS for mac (version 24) and Minitab (v18). All scripts used in the current study are available on GitHub (https://github.com/hyhy200g/MouseHypothalamus_snRNA-seq) and on Zenodo (DOI: 10.5281/zenodo.10654924).

For manuscripts utilizing custom algorithms or software that are central to the research but not yet described in published literature, software must be made available to editors and reviewers. We strongly encourage code deposition in a community repository (e.g. GitHub). See the Nature Portfolio guidelines for submitting code & software for further information.

Data

Policy information about availability of data

All manuscripts must include a data availability statement. This statement should provide the following information, where applicable:

- Accession codes, unique identifiers, or web links for publicly available datasets
- A description of any restrictions on data availability
- For clinical datasets or third party data, please ensure that the statement adheres to our policy

The raw mouse hypothalamus snRNA-seq FASTQ files generated in this study have been deposited in the Gene Expression Omnibus (GEO) database under accession code GSE217677 (https://www.ncbi.nlm.nih.gov/geo/query/acc.cgi?acc=GSE217677). The reference genome used for raw sequencing reads alignment is mm10-3.0.0. The processed mouse hypothalamus data is also available at GSE217677. Dataset related to HypoMap project used in the study are available at: https://www.repository.cam.ac.uk/items/8f9c3683-29fd-44f3-aad5-7acf5e963a75). An interactive data portal is developed based on the processed dataset and is available online https://mouse10x.shinyapps.io/p15atlas/. All experimental data supporting the findings of this study are available within the paper and its Supplementary Information, and stored as Source Data.

Research involving human participants, their data, or biological material

Policy information about studies with human participants or human data. See also policy information about sex, gender (identity/presentation), and sexual orientation and race, ethnicity and racism.

Reporting on sex and gender	N/A
Reporting on race, ethnicity, or other socially relevant groupings	N/A
Population characteristics	N/A
Recruitment	N/A
Ethics oversight	N/A

Note that full information on the approval of the study protocol must also be provided in the manuscript.

Field-specific reporting

Please select the one below that is the best fit for your research. If you are not sure, read the appropriate sections before making your selection.

☒ Life sciences ☐ Behavioural & social sciences ☐ Ecological, evolutionary & environmental sciences

For a reference copy of the document with all sections, see nature.com/documents/nr-reporting-summary-flat.pdf

Life sciences study design

All studies must disclose on these points even when the disclosure is negative.

Sample size	Initially, C57BL/6N female mice were randomly allocated to low fat diet and high fat diet on lactation day 1 (n = 10 for each group). Since female mice occasionally cull pups during lactation. We retained litters with a litter size over 5 for our experimental evaluation, which results in the final sample sizes with n=9 for maternal LFD group and n=6 for maternal HFD group. Two male or female pups were pooled for each sample (two replicates for each group, in total 8 samples) at postnatal day 15 for the collection of hypothalamus single nucleus RNA-seq analysis. For the IHC validation that in Npy-hrGFP mice, the same criteria as above were carried over in the experiment, six female mice were assigned to LFD or HFD on lactation day1, the final sample sizes included in the imaging analysis for male offspring from maternal LFD and HFD groups were 4 and 5, for female offspring from maternal LFD and HFD groups were both 5.
Data exclusions	Female mice occasionally cull pups during lactation. We previously demonstrated that low litter sizes <5 will impact the maternal lactation performance with decreased maternal food intake and milk energy output even under the HFD exposure. To remove these effects that potentially impact the outcome, we retained litters with a litter size over 5 for our experimental evaluation.
Replication	For the metabolic phenotype measurements and IHC validation, all experiments were successfully replicated with individual animals at least a minimum of three times, with the sample sizes provided above and in the manuscript. For single nucleus RNA-seq data collection, two replicates of each group were included, all attempts at replication were successful and included in the downstream analysis.
Randomization	All samples were randomly selected and assigned to each group for analysis.
Blinding	Investigators were blinded to allocate the mice to different experimental groups.

Reporting for specific materials, systems and methods

We require information from authors about some types of materials, experimental systems and methods used in many studies. Here, indicate whether each material, system or method listed is relevant to your study. If you are not sure if a list item applies to your research, read the appropriate section before selecting a response.

Materials & experimental systems

n/a	Involved in the study
☐	☒ Antibodies
☒	☐ Eukaryotic cell lines
☒	☐ Palaeontology and archaeology
☐	☒ Animals and other organisms
☒	☐ Clinical data
☒	☐ Dual use research of concern
☒	☐ Plants

Methods

n/a	Involved in the study
☒	☐ ChIP-seq
☒	☐ Flow cytometry
☒	☐ MRI-based neuroimaging

Antibodies

Antibodies used

IHC:
DAPI, Sigma-Aldrich, Cat#: D9542, 1:1000
anti-S100β, Beyotime, Cat#: AF1945, 1:500
Goat Anti-Rabbit IgG H&L (Alexa Fluor 647) preadsorbed, Abcam, Cat#: ab150087, 1:1000

Validation

1.DAPI Sigma-Aldrich Cat#: D9542
Website https://www.sigmaaldrich.cn/CN/zh/product/sigma/d9542
Species All
Applications IHC/IF
Reference:
1)Itoga CA et al. New viral-genetic mapping uncovers an enrichment of corticotropin-releasing hormone-expressing neuronal inputs to the nucleus accumbens from stress-related brain regions. J Comp Neurol 527(15):2474-2487 (2019).
2)Goldshmit Y et al. Fgf2 improves functional recovery-decreasing gliosis and increasing radial glia and neural progenitor cells after spinal cord injury. Brain Behav 4(2):187-200 (2014).
2. anti-S100β Beyotime Cat#: AF1945
Website https://www.beyotime.com/product/AF1945.htm
Species Rabbit
Applications WB, IP, IF, IHC, ICC
3. Goat Anti-Rabbit IgG H&L (Alexa Fluor 647) preadsorbed Abcam Cat#: ab150087
Website https://www.abcam.cn/products/secondary-antibodies/goat-rabbit-igg-hl-alexa-fluor-647-preadsorbed-ab150087.html
Species Goat
Applications ICC/IF, Flow Cyt, ELISA, IHC-P, IHC-Fr
Reference:
1) Welke RW et al. Characterization of Hantavirus N Protein Intracellular Dynamics and Localization. Viruses 14:N/A (2022).
2) Wang L et al. Ciliary transition zone proteins coordinate ciliary protein composition and ectosome shedding. Nat Commun 13:3997 (2022).

Animals and other research organisms

Policy information about studies involving animals; ARRIVE guidelines recommended for reporting animal research, and Sex and Gender in Research

Laboratory animals

C57BL/6N mice were purchased at 5 weeks old from Charles Rivers, Beijing and acclimated to the specific pathogen-free (SPF) facility before starting the baseline. Npy-hrGFP (#006417) with a C57BL/6J background was obtained from Jackson Laboratory and maintained at the facility afterwards. All animals were kept at 23±1°C with a dark–light cycle of 12 h–12 h (lights on at 0730 h) and under 40-70% humidity. Female mice at 11 weeks of age were fed with LFD for 2 weeks as the baseline. Mice were mated at 13 weeks old and remained on LFD until parturition. Female mice were then randomly allocated to LFD and HFD on lactation day 1.

Wild animals

No wild animals were used in this study.

Reporting on sex

Female mice at 11 weeks old were used since we were aiming to evaluate the maternal over-nutritional effect on their offspring during lactation. We fed the female mice low fat and high fat diets during lactation day 0 to day16. We further evaluated such effect on both male and female offspring.

Field-collected samples

No field-collected samples were used in this study.

Ethics oversight

All animal procedures were reviewed and approved by the animal ethical panel at Institute of Genetics and Developmental Biology, Chinese Academy of Sciences (approval number: AP2020001).

Note that full information on the approval of the study protocol must also be provided in the manuscript.
